# Association of Left Atrial Stiffness With Risk of Cryptogenic Ischemic Stroke in Young Adults

**DOI:** 10.1016/j.jacadv.2024.100903

**Published:** 2024-03-14

**Authors:** Rasmus Bach Sindre, Eva Gerdts, Jukka Putaala, Lisa M.D. Grymyr, Helga Midtbø, Ana G. Almeida, Odd Bech-Hanssen, Raila Busch, Rune K. Eilertsen, Ana Catarina Fonseca, Marja Hedman, Juha Huhtakangas, Pekka Jäkälä, Riikka Lautamäki, Mika Lehto, Nicolas Martinez-Majander, Petra Redfors, Tomi Sarkanen, Bettina von Sarnowski, Juha Sinisalo, Marko Virtanen, Ulrike Waje-Andreassen, Nilufer Yesilot, Pauli Ylikotila, Dana Cramariuc

**Affiliations:** a Department of Clinical Science, University of Bergen, Bergen, Norway; b Department of Clinical Science, Center for Research on Cardiac Disease in Women, University of Bergen, Bergen, Norway; c Department of Neurology, Helsinki University Hospital and University of Helsinki, Finland; d Department of Heart Disease, Haukeland University Hospital, Bergen, Norway; e Cardiology, Heart and Vessels Department, Faculty of Medicine of Lisbon University, University Hospital Santa Maria, Lisbon, Portugal; f Department of Clinical Physiology, Sahlgrenska University Hospital and Institute of Medicine, Sahlgrenska Academy at the University of Gothenburg, Gothenburg, Sweden; g Department of Internal Medicine B (Cardiology), University Medicine Greifswald, Greifswald, Germany; h Department of Neurosciences (Neurology), Hospital de Santa Maria, University of Lisbon, Lisbon, Portugal; i Heart Center and Clinical Imaging Center, Kuopio University Hospital and University of Eastern Finland, Kuopio, Finland; j Department of Neurology, Oulu University Hospital, Oulu, Finland; k Research Unit of Clinical Neuroscience, University of Oulu, Oulu, Finland; l Department of Neurology, Kuopio University Hospital and University of Eastern Finland, Kuopio, Finland; m Heart Centre, Turku University Hospital and University of Turku, Turku, Finland; n Department of Internal Medicine, Jorvi Hospital, HUS Helsinki University Hospital and University of Helsinki, Helsinki, Finland; o Department of Neurology, Helsinki University Hospital and University of Helsinki, Helsinki, Finland; p Department of Clinical Neuroscience, Institute of Neuroscience and Physiology, Sahlgrenska Academy, University of Gothenburg, Gothenburg, Sweden; q Department of Neurology, Sahlgrenska University Hospital, Gothenburg, Sweden; r Department of Neurology, Tampere University Hospital and Faculty of Medicine and Health Technology, Tampere University, Tampere, Finland; s Department of Neurology, University Medicine Greifswald, Greifswald, Germany; t Department of Cardiology, Heart and Lung Center, University of Helsinki and Helsinki University Hospital, Helsinki, Finland; u Heart Hospital, Tampere University Hospital, Tampere, Finland; v Department of Neurology, Haukeland University Hospital, Bergen, Norway; w Istanbul Faculty of Medicine, Department of Neurology, Istanbul University, Istanbul, Turkiye; x Neurocenter, Turku University Hospital and Clinical Neurosciences, University of Turku, Turku, Finland

**Keywords:** cryptogenic ischemic stroke, left atrial mechanics, left atrial stiffness, obesity, stroke in young adults

## Abstract

**Background:**

Incidence of cryptogenic ischemic stroke (CIS) in young adults is increasing. Early left atrial (LA) myopathy might be 1 of the underlying mechanisms, but this has only been scarcely explored.

**Objectives:**

The purpose of this study was to assess the association between increased LA stiffness and CIS in young adults.

**Methods:**

In the multicenter SECRETO (Searching for Explanations for Cryptogenic Stroke in the Young: Revealing the Etiology, Triggers, and Outcome) study, LA function was analyzed by speckle tracking echocardiography in 150 CIS patients (aged 18-49 years) and 150 age- and sex-matched controls. Minimum and maximum LA volumes, LA reservoir and contractile strain were measured. LA stiffness was calculated by the ratio: mitral peak E-wave velocity divided by mitral annular e’ velocity (E/e′)/LA reservoir strain and considered increased if ≥0.22. Increased LA volumes, LA stiffness, and/or reduced LA strain indicated LA myopathy. Logistic regression was used to determine the relation between LA stiffness and CIS and the clinical variables associated with LA stiffness.

**Results:**

Increased LA stiffness was found in 36% of patients and in 18% of controls (*P* < 0.001). Increased LA stiffness was associated with a 2.4-fold (95% CI: 1.1-5.3) higher risk of CIS after adjustment for age, sex, comorbidities, and echocardiographic confounders (*P* = 0.03). In patients, obesity, pre-CIS antihypertensive treatment, older age, and lower LA contractile strain were all related to increased LA stiffness (all *P* < 0.05).

**Conclusions:**

LA myopathy with increased LA stiffness and impaired LA mechanics more than doubles the risk of CIS in patients under the age of 50 years. This provides new insights into the link between LA dysfunction and CIS at young ages. (Searching for Explanations for Cryptogenic Stroke in the Young: Revealing the Etiology, Triggers, and Outcome [SECRETO]; NCT01934725)

Premature ischemic stroke affects over 1.5 million young adults (aged 18-49 years) annually, is increasing in prevalence in high-income countries and is classified as of undetermined cause or cryptogenic ischemic stroke (CIS) in up to 50% of cases.^1^ Young stroke survivors have a high long-term rate of subsequent cardiovascular (CV) adverse events and stand for almost half of the entire stroke burden globally.^2^, ^3^, ^4^

The etiological exploration of CIS in young patients has previously focused on the role of coagulopathies and of patent foramen ovale (PFO) as potential sources of paradoxical embolism.^5^ The evidence for a possible causal relationship between these conditions and CIS remains however poor. A previous meta-analysis showed that the rate of PFO-related CIS was as low as 1%/year, and percutaneous PFO closure in highly selected individuals resulted in only a modest benefit.^6^ Atrial fibrillation is uncommon in young adults, and hence most CIS in young adults are thought not to be due to silent atrial fibrillation.^7^ The prevalence of traditional vascular risk factors is increasing among young adults, but this has not translated into a more frequent identification of classical stroke mechanisms in young stroke patients.^8^

More recently, impaired left atrial (LA) strain has been linked to higher risk of ischemic stroke in older patients after taking into account LA size and incident atrial fibrillation, and to recurrent CIS in the sixth to eighth decade of life.^9^^,^^10^ Preliminary data on 30 CIS patients and 30 controls from the SECRETO (Searching for Explanations for Cryptogenic Stroke in the Young: Revealing the Etiology, Triggers, and Outcome) study have indicated that LA mechanics might be altered in young CIS patients.^11^ By cardiac magnetic resonance imaging, elderly patients with stroke of undetermined etiology have been shown to have more LA fibrosis than those with strokes of other causes, suggesting atrial myopathy as a substrate for CIS.^12^ An atrial myocardial remodeling process with accumulating wall fibrosis in response to various stimuli might start earlier in life and increase the chance of ischemic events. This has not been previously explored in young CIS patients.

The current study aimed to analyze the association between increased LA stiffness and risk of stroke in a European cohort of young adults hospitalized with first-ever CIS and included in the SECRETO study. Moreover, we sought to identify the clinical and echocardiographic factors specifically associated with increased LA stiffness in young CIS adults.

## Methods

### Study design

SECRETO is an international prospective, multicenter, case-control study of young adults (aged 18-49 years) hospitalized due to first-ever imaging-proven acute CIS. The study rationale and design have been previously published.^13^ In short, patients were included at 19 study centers in the period 2013 to 2022 after extensive diagnostic work-up including brain magnetic resonance imaging, imaging of intracranial and extracranial vessels, echocardiography, electrocardiogram (ECG) recordings of at least 24 hours for detection of atrial arrhythmias, and screening for coagulopathies. Strokes that remained of unknown cause, uncertain cause, or with no likely direct cause after these investigations were classified as CIS.^13^ To identify risk factors linked to CIS in patients as opposed to the general population, each patient was matched to 1 stroke-free control of similar age, sex, and ethnicity identified in the community and included at the same study center. Patients with other strokes than CIS and those in whom minimum diagnostic tests (eg, brain magnetic resonance imaging, blood tests) were not performed during the first week, or other predefined SECRETO diagnostic tests were not performed during the first 2 weeks after stroke, were excluded. For the present analysis, to ensure a power of 80% at alpha error 0.05, LA stiffness and function were assessed and compared in the first 150 patients and 150 matched controls recruited in SECRETO.

Based on previous data linking abdominal obesity to increased risk of CIS, we assessed body size by both waist and hip circumference, waist-to-hip ratio, and body mass index (BMI).^14^ Patients were categorized as obese if their BMI was ≥30 kg/m^2^, and abdominally obese if the waist-to-hip ratio exceeded 0.85 in women and 0.90 in men.^14^ Hypertension was defined as blood pressure ≥140/90 mmHg at the study visit, history of hypertension, and/or use of antihypertensive drugs prior to CIS. Diabetes mellitus was present if the patient/control had high fasting blood sugar, previously diagnosed diabetes or used antidiabetic medication. Tobacco use included previous and current cigarette smoking. Physical inactivity (activity less-than-moderate) was assessed by the short version of the International Physical Activity Questionnaire.^15^ Heavy alcohol consumption was defined as more than 5 U of alcohol/day or 16 U/week for women, and 7 U of alcohol/day or 24 U/week for men.^16^ Dietary habits were evaluated by a modified Mediterranean diet score.^17^

The SECRETO study was approved by the ethics committees of all participating centers, and all participants provided written informed consent.

### Echocardiographic measurements

Echocardiography was performed at all study centers using a standardized protocol.^18^ The echocardiograms were forwarded in a Digital Imaging and Communications in Medicine format and analyzed by a junior and proofread by a senior reader at the SECRETO echo core laboratory at Haukeland University Hospital, Bergen, Norway.

#### LA size, function, and stiffness

Minimum, pre-A, and maximum LA volumes (LAVmax) were measured in dedicated, non-foreshortened apical 4- and 2- chamber views at end-diastole, onset of atrial contraction (identified by the P-wave on ECG) and end-systole, respectively.^19^ LA appendage and the pulmonary veins ostia were excluded from the volumes. The LA reservoir volume was calculated as the difference between maximum and minimum LA volume (LAVmin).^20^ LA strain was measured using a vendor independent 2D speckle tracking software (2D Cardiac Performance Analysis, Tomtec Arena, Germany) with end-diastole as the zero-strain reference point.^20^ LA reservoir function was assessed by the LA reservoir strain (LASr) and the LA reservoir work (LAWr), and LA pump function by the LA contractile strain (LASct). LAWr was deducted from the product LASr x LA reservoir volume as previously described.^21^ The ratio of early mitral inflow velocity and mitral annular early diastolic velocity, that is, the mitral E/e′ was used to estimate the left-sided filling pressure.^22^ Mitral e′ was averaged from the septal and lateral values. LA stiffness was further calculated as the ratio of mitral E/e′ to LASr, a method previously validated against invasive measurements.^23^ LA stiffness above 0.22 was considered increased, representing the upper normal limit of E/e′/LASr in young healthy European subjects.^24^ LA myopathy was considered present if LA volumes and/or stiffness were increased, or if LA mechanics assessed by either LASr, LAWr, or LASct was reduced.^25^

#### Left ventricular size and function

Left ventricular (LV) hypertrophy was defined as LV mass indexed for height^2.7^ ≥49.2 g/m^2.7^ in men and ≥46.7 g/m^2.7^ in women, as recommended in populations with higher prevalence of obesity.^26^ The relative wall thickness was deducted from the ratio of end-diastolic LV posterior wall thickness to the LV internal diameter. Global LV systolic function was measured by Simpson’s biplane ejection fraction (EF, low if <52% in men and <54% in women),^27^ and LV long-axis function by the mitral annular peak systolic velocity (ś) using tissue Doppler imaging. LV pump performance was assessed by the Doppler stroke volume. Systemic arterial compliance was estimated from the Doppler stroke volume index divided by the pulse pressure.

### Biochemical analyses

Blood samples were collected in all patients at study inclusion and used for measurement of serum creatinine, lipid profile, and glycated hemoglobin A_1c_.

### Statistical analyses

Statistical analyses were performed in IBM SPSS Statistics 28.0 (IBM Corp) as well as R (R core team, 2023, R studio, Posit Team, 2023 and the ggplot2 package, Wickham, 2016). All clinical and echocardiographic variables were compared between patients and controls.

Normality was evaluated with both Shapiro-Wilk and Q-Q plots. For normally distributed continuous variables, groups were compared using independent samples and paired samples *t*-tests. When data were not normally distributed, correlations were assessed by the Spearman test, and differences between groups tested by bootstrap *t*-tests. Categorical variables were compared by the chi-square test. Findings are reported as mean ± SD or as percentages.

The relation between clinical and echocardiographic findings and occurrence of CIS was assessed in the whole population using conditional regressions analyses. Clinically relevant variables and variables associated with CIS at a 2-sided probability <0.1 in univariable analyses were selected into multivariable conditional logistic regression models testing the association between LA stiffness and CIS. In the first model, the association was adjusted for age and sex. In subsequent models, additional adjustment for relevant comorbidities and then echocardiographic findings was performed.

In patients, factors associated with increased LA stiffness at a *P* value <0.10 in univariable logistic regression analyses were further tested in multivariable logistic regression analysis. The adjusted ORs with 95% CIs are presented in a forest plot. All logistic multivariable models were evaluated by the 2 log likelihood and the Nagelkerke R^2^ value. A 2-tailed *P* < 0.05 was considered significant in all analyses.

## Results

### Clinical characteristics of patients with CIS and controls in relation to LA stiffness

**Figure 1:**
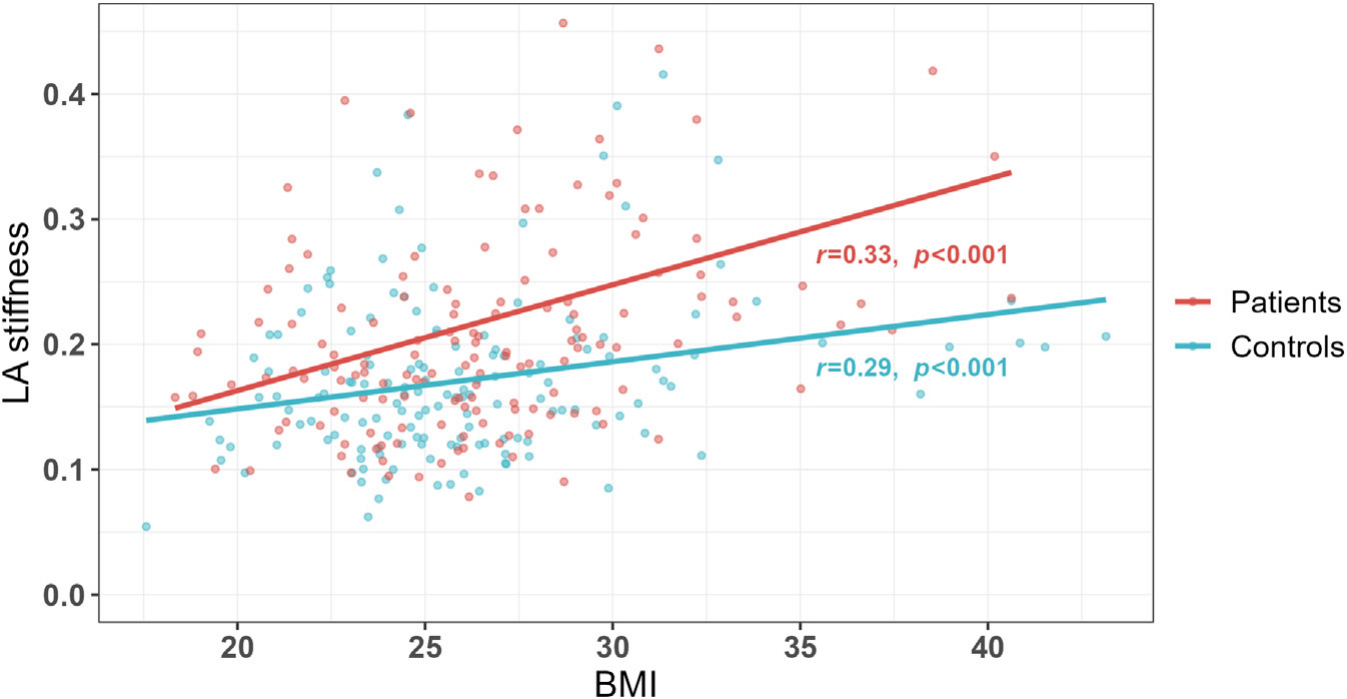
**Scatter Plots of Body Mass Index vs Left Atrial Stiffness in Patients With Cryptogenic Ischemic Stroke and Controls** The correlation is assessed in each group by the correlation coefficient (r) with *P* level of significance. BMI = body mass index; LA = left atrial.

Among young adults hospitalized with first-ever CIS, an increased LA stiffness was identified in 36% compared to 18% of the matched controls (*P* < 0.001). No study participant had a history of CV disease, and diabetes was uncommon (n = 3). Both patients and controls with increased LA stiffness were on average 5 years older and had higher heart rate and higher prevalence of obesity than subjects with normal LA stiffness (all *P* < 0.05) (Table 1). However, at each BMI value, LA stiffness was higher in patients than in controls (Figure 1). In patients with stiffer LA, waist circumference and waist-to-hip ratio were larger, and hypertension and antihypertensive medication significantly more common before hospitalization than in both controls and patients with normal LA stiffness (Table 1).

**Table 1:** Anthropometrics and Clinical Characteristics of Patients With Cryptogenic Ischemic Stroke Compared to Controls

	CIS Patients (n = 150)			Controls (n = 150)		
	Increased LA Stiffness (n = 54)	Normal LA Stiffness (n = 96)	*P* Value	Increased LA Stiffness (n = 27)	Normal LA Stiffness (n = 123)	*P* Value
Age, y	42 ± 6	37 ± 8	**<0.001**^a^	43 ± 7	38 ± 8	**0.004**^a^
Women	43%	48%	0.609	59%	44%	0.201
Waist, cm	99 ± 15	89 ± 12	**<0.001**^a^	94 ± 16	89 ± 12	0.249^a^
Waist-to-hip ratio	0.93 ± 0.10‡	0.88 ± 0.08	**0.003**^a^	0.87 ± 0.09	0.87 ± 0.09	0.740^a^
BMI, kg/m^2^	28.7 ± 4.7	25.5 ± 3.5	**<0.001**^a^	28.2 ± 7.6	25.9 ± 4.3	0.175^a^
Overweight	41%‡	47%	**<0.001**	15%	42%	**0.003**
Obesity	37%	7%	**<0.001**	33%	11%	**0.003**
Heart rate, beats/min	72 ± 13	66 ± 12‡	**0.023**^a^	69 ± 11	62 ± 16	**0.005**^a^
Systolic BP, mm Hg	127 ± 15	122 ± 13†	0.062^a^	132 ± 15	128 ± 13	0.192^a^
Diastolic BP, mm Hg	78 ± 10	72 ± 10†	**0.002**^a^	83 ± 13	78 ± 11	0.071^a^
Hypertension	51%‡	29%	**0.008**	39%	27%	0.230
Pre-CIS treatment						
Antihypertensive treatment	17%	4%	**0.008**	9%	7%	0.760
Lipid-lowering treatment	4%	0%	Na	4%	2%	0.429
Ex-smokers	21%	23%	0.456	30%	19%	**0.045**
Physical inactivity	12%	7%	0.395	4%	5%	0.856
Heavy alcohol consumption	25%	19%	0.405	22%	10%	0.092

Values are mean ± SD or %. *P* values indicate the level of significance when comparing patients with increased vs normal LA stiffness and controls with increased vs normal LA stiffness, respectively (**bold** indicates *P* < 0.05).

†*P* < 0.01 and ‡*P* < 0.05 when comparing patients with controls in either the group with increased LA stiffness or the group with normal LA stiffness.

BMI = body mass index; BP = blood pressure; CIS = cryptogenic ischemic stroke; LA = left atrial.

a Indicates comparison by bootstrap *t*-tests.

Overall, patients had an unhealthier lifestyle with less regular intake of legumes (<2 servings/week in 49% vs 37%) and higher prevalence of tobacco smoking (56% vs 42%) and heavy alcohol consumption (21% vs 12%) (all *P* < 0.05) (Supplemental Table). While dietary habits and physical inactivity were not associated with LA stiffness, previous smoking was more common among controls with increased LA stiffness, and heavy drinking numerically more frequent in subjects with stiffer LA (Table 1). Average serum creatinine (73 vs 71 μmol/L) and total cholesterol (7.7 vs 6.2 mmol/L) were comparable between patients with vs without increased LA stiffness and not associated with LA stiffness in univariable regression analyses.

### LA function and stiffness in patients and controls

**Figure 2:**
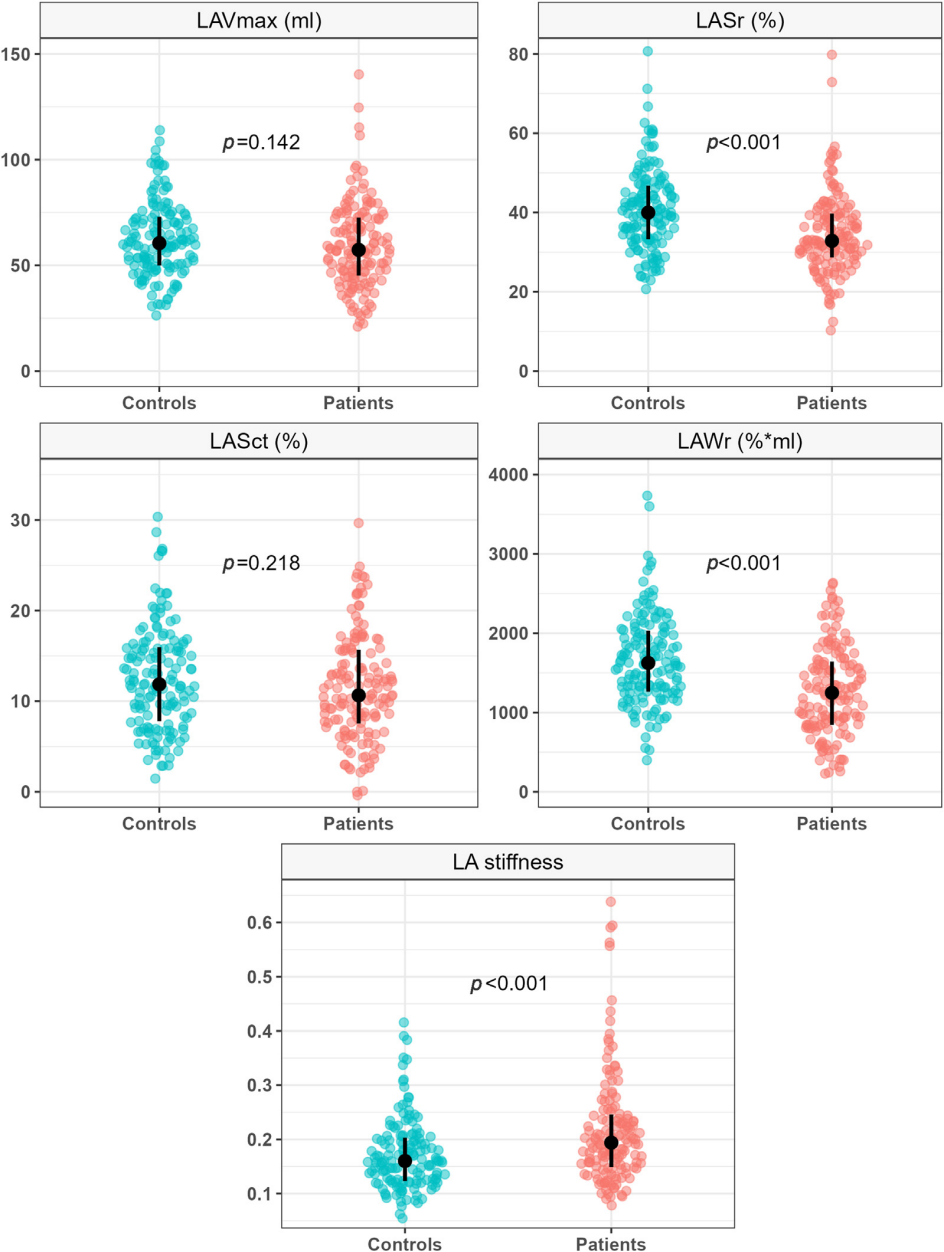
**Comparison of Maximum Left Atrial Volume, Left Atrial Reservoir Strain, Left Atrial Contractile Strain, Left Atrial Reservoir Work, and Left Atrial Stiffness Between Patients With Cryptogenic Ischemic Stroke and Controls** Comparisons were made by bootstrap *t*-tests with *P* level of significance. The distribution of each variable is presented as a violin plot with the median and interquartile range marked in black bars. LA = left atrial; LASct = left atrial contractile strain; LASr = left atrial reservoir strain; LAVmax = maximum left atrial volume; LAWr = left atrial reservoir work.

CIS patients had more impaired LA reservoir function and increased LA stiffness compared to controls (Figure 2). Both patients and controls with stiff LA had larger LAVmin, lower LASr and LAWr, and decreased LA pump function by LASct (all *P* < 0.01) (Table 2). Patients with increased LA stiffness had the lowest level of reservoir function of the whole study population.

**Table 2:** Cardiac Size and Function in Patients With Cryptogenic Ischemic Stroke Compared to Controls

	CIS Patients (n = 150)			Controls (n = 150)		
	Increased LA Stiffness (n = 54)	Normal LA Stiffness (n = 96)	*P* Value	Increased LA Stiffness (n = 27)	Normal LA Stiffness (n = 123)	*P* Value
LAVmax, mL	63 ± 23	57 ± 18	0.125^a^	71 ± 19	61 ± 17	**0.016**^a^
LAVmax/BSA, mL/m^2^	31 ± 10	30 ± 9	0.436^a^	36 ± 10	31 ± 8	**0.029**^a^
LAVmin, mL	27 ± 14	20 ± 8	**<0.001**^a^	27 ± 8	20 ± 8	**<0.001**^a^
LASr, %	27.1 ± 6.3	38.7 ± 9.4∗	**<0.001**^a^	29.9 ± 5.4	43.1 ± 9.5	**<0.001**^a^
LASct, %	10.0 ± 4.3	12.6 ± 6.9	**0.006**	9.7 ± 4.7	13.1 ± 5.8	**0.002**
LAWr, % × mL	987 ± 461†	1,443 ± 545∗	**<0.001**^a^	1,322 ± 516	1,763 ± 593	**<0.001**^a^
Mitral s′	8.4 ± 1.6	9.3 ± 1.7	**0.002**	8.5 ± 1.2	9.2 ± 1.6	**0.036**
Mitral E/e′	8 ± 2	6 ± 1	**<0.001**^a^	8 ± 2	6 ± 1	**<0.001**^a^
LA stiffness	0.32 ± 0.12‡	0.16 ± 0.04‡	Na	0.28 ± 0.06	0.15 ± 0.04	Na
LV end-diastolic volume, mL	137 ± 41	127 ± 34	0.142^a^	133 ± 34	135 ± 31	0.759^a^
LV end-systolic volume, mL	63 ± 22	56 ± 18	0.077^a^	59 ± 20	59 ± 16	0.998^a^
LV hypertrophy	14%	4%	**0.030**	4%	0%	Na
Relative wall thickness	0.30 ± 0.09	0.26 ± 0.06	**0.013**^a^	0.28 ± 0.06	0.26 ± 0.06	0.104^a^
LV EF, %	55 ± 6	56 ± 5	0.134	56 ± 5	57 ± 4	0.757
Right ventricular basal diameter, cm	35 ± 7	34 ± 6	0.522	33 ± 4	34 ± 5	0.227
TAPSE, mm	25 ± 4	26 ± 5	0.520	25 ± 1	27 ± 3	0.446
Systemic arterial compliance, mL/m^2^/mm Hg	0.78 ± 0.26	0.87 ± 0.24	**0.037**^a^	0.87 ± 0.24	0.85 ± 0.23	0.749^a^

Values are mean ± SD or %. *P* values in indicate the level of significance when comparing patients with increased vs normal LA stiffness and controls with increased vs normal LA stiffness, respectively (**bold** indicates *P* < 0.05).

∗*P* < 0.001, †*P* < 0.01 and ‡*P* < 0.05 when comparing patients and controls in either the group with increased LA stiffness or the group with normal LA stiffness.

BSA = body surface area; EF = ejection fraction; LA = left atrial; LASct = left atrial contractile strain; LASr = left atrial reservoir strain; LAVmax = maximum left atrial volume; LAVmin = minimum left atrial volume; LAWr = left atrial reservoir work; LV = left ventricle; TAPSE = tricuspid annulus peak systolic excursion.

a Indicates comparison by bootstrap *t*-tests.

LV volumes and right ventricular size did not differ between groups. However, LV relative wall thickness was increased, and LV hypertrophy more prevalent in patients with increased LA stiffness (Table 2). None of the patients had apical or non-apical ballooning as in stress cardiomyopathy, and the average EF was normal in patients and controls (56 ± 5 vs 57 ± 5) and not different between LA stiffness groups. Patients with increased LA stiffness had reduced systemic arterial compliance (Table 2).

In conditional regression analysis, increased LA stiffness was associated with 2.5-fold higher odds of CIS after adjustment for age and sex (Table 3). After further adjustment for relevant comorbidities (abdominal obesity, hypertension, use of antihypertensive medication, heavy alcohol consumption, and tobacco use) and echocardiographic factors (LV mass, relative wall thickness, LVEF, and systemic arterial compliance), increased LA stiffness retained a strong association with CIS with an OR of 2.4 (Table 3). When LAVmax was forced into the model, the results remained unchanged. In similar models, decreased LA reservoir dynamics assessed by either LASr or LAWr, and increased LAVmax were also associated with risk of CIS after multiple adjustments, while LAVmin and LA pump function failed to reach statistical significance (Table 3). Of note, mitral s′ was not associated with CIS in univariable analysis (*P* = 0.55) and consequently not further included in multivariable models.

**Table 3:** Association Between Left Atrial Size, Function, and Stiffness and Cryptogenic Ischemic Stroke in the Entire Cohort

	Model 1 (Adjustment for Age and Sex)^a^		Model 2 (Adjustment for Comorbidities)^b^		Model 3 (Adjustment for Comorbidities and Echo Variables)^c^	
	OR (95% CI)	*P* Value	OR (95% CI)	*P* Value	OR (95% CI)	*P* Value
LAVmax, mL	0.99 (0.90-1.01)	0.335	0.98 (0.97-1.00)	**0.034**	0.98 (0.96-1.00)	**0.033**
LAVmin, mL	1.02 (1.00-1.05)	0.107				
LASr, %	0.95 (0.92-0.98)	**<0.001**	0.95 (0.93-0.98)	**0.001**	0.95 (0.92-0.99)	**0.008**
LASct, %	0.99 (0.95-1.04)	0.690				
LAWr, % × mL	0.999 (0.998-0.999)	**<0.001**	0.999 (0.998-0.999)	**<0.001**	0.998 (0.998-0.999)	**<0.001**
Increased LA stiffness	2.53 (1.38-4.63)	**0.003**	2.34 (1.19-4.60)	**0.014**	2.39 (1.07-5.33)	**0.033**

**Bold** indicates *P* < 0.05.

LA = left atrial; LASct = left atrial contractile strain; LASr = left atrial reservoir strain; LAVmax = maximum left atrial volume; LAVmin = minimum left atrial volume; LAWr = left atrial reservoir work.

a Model 1 included stratification by age and sex.

b Model 2 included adjustment for age, sex, and the following comorbidities: abdominal obesity, hypertension, use of antihypertensive medication, heavy alcohol consumption, and tobacco use.

c Model 3 included adjustment for sex, age, the comorbidities included in model 2, and the following echo variables: LV hypertrophy, LV relative wall thickness, LV EF, and systemic arterial compliance.

### Factors associated with increased LA stiffness in young CIS patients

In multivariable logistic regression analysis, after backward stepwise selection of significant covariables among age, obesity, hypertension, use of antihypertensive medication, tobacco or heavy alcohol consumption, LV hypertrophy, EF, systemic arterial compliance and LASct, obesity (12.4-fold higher odds), and use of antihypertensive medication prior to CIS (5.8-fold higher odds) were strongly associated with stiffer LA in patients (−2 log likelihood 133, Nagelkerke R^2^ 0.40) (Central Illustration). Higher age and lower LASct were also significantly and independently related to increased LA stiffness (Central Illustration). When the same variables were tested in relation to LA stiffness in a linear regression analysis, 30% of the variation in LA stiffness in young CIS patients could be explained by this model (R^2^ = 0.30, *P* < 0.001). Finally, replacing BMI with abdominal obesity, the latter was also related to increased LA stiffness but had a lower OR value of 2.4 (95% CI: 1.0-5.7, *P* < 0.05).

**Central Illustration:**
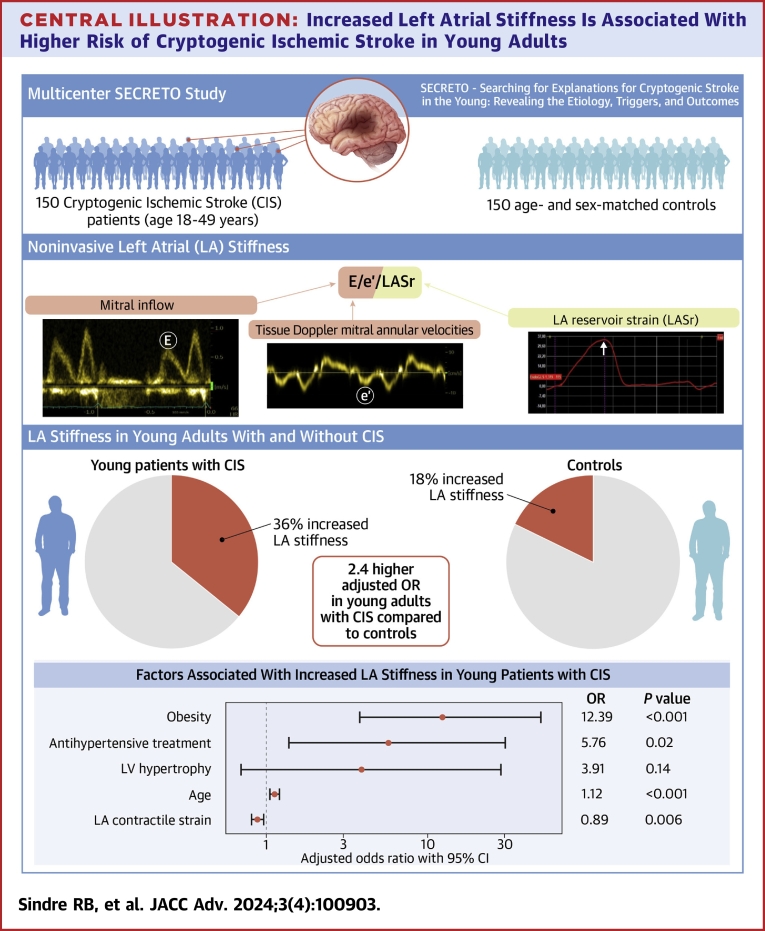
**Increased Left Atrial Stiffness Is Associated With Higher Risk of Cryptogenic Ischemic Stroke in Young Adults** E = early mitral inflow velocity; e′ = mitral annular early diastolic velocity; LV = left ventricular.

## Discussion

Ischemic stroke in young adults is increasingly prevalent in high-income countries and remains often of uncertain etiology despite modern-day diagnostic work-up. Using case-control data from the prospective SECRETO study, we investigated whether premature LA disease with increased LA stiffness and impaired LA dynamics is one of the mechanisms underlying CIS in adults aged 18 to 49 years. Our analysis showed that increased LA stiffness was twice as common in CIS patients as in age- and sex-matched controls, being present in over one-third of patients. Furthermore, increased LA stiffness was associated with 2.4-fold higher odds of CIS in our population after adjustment for demographics, comorbidities, and echocardiographic findings. Finally, obesity and treatment for hypertension prior to CIS were strongly related to excessive LA stiffness among young CIS patients, underscoring the importance of strict CV risk control at younger ages to prevent ischemic stroke.

### LA size and function in stroke patients

LA remodeling has been recognized early as a risk factor for both incident and recurrent stroke.^28^ Most of the earlier research has focused on the causal relationship between LA enlargement and cerebrovascular disease, a pathway at least partially mediated by atrial fibrillation. However, LA must be at least moderately enlarged to predict recurrent stroke, a stage usually characterized by advanced remodeling with extensive structural changes in the LA myocardium.^28^^,^^29^ In young ischemic stroke survivors, LAVmax is often within the normal range, as in our cohort, and atrial fibrillation a rare finding.

Recently, impaired LA dynamics with reduced reservoir strain was shown to predict the risk of ischemic stroke in a community-based cohort of adults aged 55 years or older.^9^ This relationship was independent of incident atrial fibrillation and significant even in patients with normal LA size.^9^ In a separate cohort of 5,461 individuals participating in the ARIC (Atherosclerosis Risk In Communities) study, the associations of atrial fibrillation with ischemic stroke and dementia were no longer significant after adjustment for measures of LA myopathy and in particular for LA reservoir strain.^30^ In older CIS patients (average age of 63 years), both LASr and LASct have been identified as predictors of 2-year risk of recurrent stroke after adjustment for kidney function and smoking.^10^ A small pilot study including 30 patients and controls enrolled in the SECRETO study has earlier suggested that impaired LA wall mechanics might contribute to CIS in young adults as well.^11^ The present study builds on this observation and demonstrates in a larger cohort that LA function in the reservoir phase, assessed both as strain and work, is reduced in young CIS patients despite normal and comparable LA volumes with age- and sex-matched controls. Our findings confirm that speckle tracking-based measures of LA function detect earlier stages of LA disease than LA volumes alone. Interestingly, a pattern of impaired LA reservoir function with preserved LA pump function has earlier been described in patients with metabolic syndrome as well as in a hypertensive cohort.^31^^,^^32^ LASct was not independently associated with CIS in our study, revealing impaired LA extensibility in the reservoir phase as a more sensitive marker of LA myopathy than reduced LA pump function in CIS patients below 50 years of age.

### LA stiffness and CIS

Impaired LASr can be due to reduced preload or increased LA wall stiffness, limiting LA’s extension in response to increased LA pressure. By cardiac magnetic resonance imaging, a higher percentage of atrial fibrosis was found in 52 patients with stroke of undetermined cause compared to a group of 42 patients with stroke of a specific cause.^12^ Kurt et al^23^ demonstrated using right heart catheterization and echocardiography in combination that the ratio of E/e′/LASr is a reliable estimate of LA stiffness and correlates well with invasive measurements. We demonstrate for the first time that increased LA stiffness carries a 2.4-fold higher risk of CIS after adjustment for known vascular risk factors, LA size, and LV hypertrophy and function. Interestingly, only 22% of patients were of normal weight, and obesity (both general and abdominal) was strongly related to increased LA stiffness among patients. Obesity is also associated with chronic low-grade inflammation and impaired fibrinolysis suggesting that the combination of a stiffer, less expandable LA favorizing stasis and activation of prothrombotic pathways may lead to CIS in young obese individuals. Furthermore, we show that combined obesity and hypertension, and the associated reduced systemic arterial compliance, not only promote adverse LV remodeling but also result in more severe LA myopathy.^26^ Nevertheless, it is worth noting that 70% of the variation in LA stiffness remains unexplained by the traditional CV risk factors tested in our models, indicating that other factors not measured in the present study may contribute considerably to increased LA stiffness and, subsequently, CIS at younger age.

### Clinical implications

Young patients with CIS account for up to 50% of all premature ischemic strokes and have a high rate of stroke recurrence. Improved risk stratification and decision-making in these patients are necessary to prevent new CV events and reduce the global stroke burden. Our findings demonstrate an association between subclinical LA disease and increased ischemic stroke risk in young individuals, in particular when they present obesity and hypertension. This highlights the importance of public health policies that promote healthy lifestyles and the avoidance of vascular risk factors from an early age. Moreover, the significantly increased risk of CIS associated with stiffer LA, independent of LA size and in the absence of atrial fibrillation, suggests that patients with increased LA stiffness and diminished LA mechanics may benefit from early intervention, like antithrombotic therapy. This should be further investigated in clinical studies addressing preventive and therapeutic interventions in young patients at high risk of ischemic stroke. Reduced LA strain early after CIS might also help identify individuals at higher likelihood of developing atrial fibrillation at follow-up and in whom extended rhythm monitoring might be indicated.^33^, ^34^, ^35^ Finally, future studies should also address long-term changes in LA function and stiffness after CIS and assess how these might affect prognosis.

### Study limitations

The importance of increased LA stiffness has, to our knowledge, not been analyzed in relation to CIS before, and the results need confirmation from other CIS cohorts. The noninvasively estimated LA stiffness used in this study has not been previously validated against cardiac magnetic resonance assessment of LA fibrosis using late gadolinium enhancement. However, limited data using magnetic resonance confirm an increased profibrotic remodeling process in CIS patients. Even if most CIS in young adults are thought not to be due to silent atrial fibrillation due to its low prevalence in this age group, we acknowledge that some episodes of atrial fibrillation might not have been captured by the standard ECG monitoring of minimum 24 hours. The age range of our patients (18-49 years) was broad; however, 86% of the patients were ≥30 years old, making the results most representative for this age group. Finally, the predictive value of increased LA stiffness should also be tested in longitudinal studies to determine if this novel measure is associated with higher risk of recurrent ischemic stroke and mortality.

## Conclusions

LA myopathy with increased LA stiffness and impaired LA mechanics more than doubles the risk of CIS in patients under the age of 50 years. LA myopathy is particularly common in young stroke patients with concomitant obesity or hypertension. The findings provide new insights into a potential mechanistic link between LA dysfunction and CIS at young ages.

## Funding support and author disclosures

The SECRETO study has been funded by the 10.13039/501100002341Academy of Finland under grant numbers 318075 and 322656, Helsinki and Uusimaa Hospital District (TYH2018318), and 10.13039/501100006306Sigrid Juselius Foundation. Dr Cramariuc has received clinical researcher funds and open project support from the Regional Health Authorities in Western Norway (project numbers F-12557 and F-12615). Dr Sarkanen has received grant number 322663 from the Academy of Finland. The authors have reported that they have no relationships relevant to the contents of this paper to disclose.

PERSPECTIVES **COMPETENCY IN MEDICAL KNOWLEDGE:** Young adults with increased LA stiffness have significantly higher risk of CIS independent of LA size and in the absence of atrial fibrillation.

**TRANSLATIONAL OUTLOOK:** Patients with premature LA disease may benefit from early intervention, like antithrombotic therapy. This should be further investigated in clinical studies addressing preventive and therapeutic interventions in young patients at high risk of ischemic stroke.

## References

[1] Ekker M.S., Verhoeven J.I., Vaartjes I., van Nieuwenhuizen K.M., Klijn C.J.M., de Leeuw F.E. (2019). Stroke incidence in young adults according to age, subtype, sex, and time trends. Neurology.

[2] Krishnamurthi R.V., Moran A.E., Feigin V.L. (2015). Stroke prevalence, mortality and disability-adjusted life years in adults aged 20-64 years in 1990-2013: data from the global burden of disease 2013 study. Neuroepidemiology.

[3] Edwards J.D., Kapral M.K., Lindsay M.P., Fang J., Swartz R.H. (2019). Young stroke survivors with no early recurrence at high long-term risk of adverse outcomes. J Am Heart Assoc.

[4] Rutten-Jacobs L.C., Arntz R.M., Maaijwee N.A. (2013). Long-term mortality after stroke among adults aged 18 to 50 years. JAMA.

[5] Bhatt N., Malik A.M., Chaturvedi S. (2018). Stroke in young adults: five new things. Neurol Clin Pract.

[6] Kent D.M., Dahabreh I.J., Ruthazer R. (2016). Device closure of patent foramen ovale after stroke: pooled analysis of completed randomized trials. J Am Coll Cardiol.

[7] Lehto M., Haukka J., Aro A. (2022). Comprehensive nationwide incidence and prevalence trends of atrial fibrillation in Finland. Open Heart.

[8] George M.G., Tong X., Bowman B.A. (2017). Prevalence of cardiovascular risk factors and strokes in younger adults. JAMA Neurol.

[9] Mannina C., Ito K., Jin Z. (2023). Association of left atrial strain with ischemic stroke risk in older adults. JAMA Cardiol.

[10] Bhat A., Chen H.H.L., Khanna S. (2022). Diagnostic and prognostic value of left atrial function in identification of cardioembolism and prediction of outcomes in patients with cryptogenic stroke. J Am Soc Echocardiogr.

[11] Pirinen J., Jarvinen V., Martinez-Majander N., Sinisalo J., Poyhonen P., Putaala J. (2020). Left atrial dynamics is altered in young adults with cryptogenic ischemic stroke: a case-control study utilizing advanced echocardiography. J Am Heart Assoc.

[12] Fonseca A.C., Alves P., Inacio N. (2018). Patients with undetermined stroke have increased atrial fibrosis: a cardiac magnetic resonance imaging study. Stroke.

[13] Putaala J., Martinez-Majander N., Saeed S. (2017). Searching for explanations for cryptogenic stroke in the young: revealing the triggers, causes, and outcome (SECRETO): rationale and design. Eur Stroke J.

[14] Jaakonmaki N., Zedde M., Sarkanen T. (2022). Obesity and the risk of cryptogenic ischemic stroke in young adults. J Stroke Cerebrovasc Dis.

[15] Craig C.L., Marshall A.L., Sjostrom M. (2003). International physical activity questionnaire: 12-country reliability and validity. Med Sci Sports Exerc.

[16] Niemela O., Niemela M., Bloigu R., Aalto M., Laatikainen T. (2017). Where should the safe limits of alcohol consumption stand in light of liver enzyme abnormalities in alcohol consumers?. PLoS One.

[17] Panagiotakos D.B., Pitsavos C., Arvaniti F., Stefanadis C. (2007). Adherence to the Mediterranean food pattern predicts the prevalence of hypertension, hypercholesterolemia, diabetes and obesity, among healthy adults; the accuracy of the MedDietScore. Prev Med.

[18] Saeed S., Gerdts E., Waje-Andreassen U., Sinisalo J., Putaala J. (2019). Searching for explanations for cryptogenic stroke in the young: revealing the etiology, triggers, and outcome (SECRETO): echocardiography performance protocol. Echo Res Pract.

[19] Thomas L., Muraru D., Popescu B.A. (2020). Evaluation of left atrial size and function: relevance for clinical practice. J Am Soc Echocardiogr.

[20] Badano L.P., Kolias T.J., Muraru D. (2018). Standardization of left atrial, right ventricular, and right atrial deformation imaging using two-dimensional speckle tracking echocardiography: a consensus document of the EACVI/ASE/Industry Task Force to standardize deformation imaging. Eur Heart J Cardiovasc Imaging.

[21] Cramariuc D., Alfraidi H., Nagata Y. (2023). Atrial dysfunction in significant atrial functional mitral regurgitation: phenotypes and prognostic implications. Circ Cardiovasc Imaging.

[22] Nagueh S.F., Smiseth O.A., Appleton C.P. (2016). Recommendations for the evaluation of left ventricular diastolic function by echocardiography: an update from the American Society of Echocardiography and the European Association of Cardiovascular Imaging. J Am Soc Echocardiogr.

[23] Kurt M., Wang J., Torre-Amione G., Nagueh S.F. (2009). Left atrial function in diastolic heart failure. Circ Cardiovasc Imaging.

[24] Sugimoto T., Robinet S., Dulgheru R. (2018). Echocardiographic reference ranges for normal left atrial function parameters: results from the EACVI NORRE study. Eur Heart J Cardiovasc Imaging.

[25] Reddy Y.N.V., Obokata M., Verbrugge F.H., Lin G., Borlaug B.A. (2020). Atrial dysfunction in patients with heart failure with preserved ejection fraction and atrial fibrillation. J Am Coll Cardiol.

[26] Grymyr L.M.D., Nadirpour S., Gerdts E. (2021). Left ventricular myocardial oxygen demand and subclinical dysfunction in patients with severe obesity referred for bariatric surgery. Nutr Metab Cardiovasc Dis.

[27] Lang R.M., Badano L.P., Mor-Avi V. (2015). Recommendations for cardiac chamber quantification by echocardiography in adults: an update from the American Society of Echocardiography and the European Association of Cardiovascular Imaging. Eur Heart J Cardiovasc Imaging.

[28] Yaghi S., Moon Y.P., Mora-McLaughlin C. (2015). Left atrial enlargement and stroke recurrence: the Northern Manhattan Stroke Study. Stroke.

[29] Yaghi S., Song C., Gray W.A., Furie K.L., Elkind M.S., Kamel H. (2015). Left atrial appendage function and stroke risk. Stroke.

[30] Zhang M.J., Ji Y., Wang W. (2023). Association of atrial fibrillation with stroke and dementia accounting for left atrial function and size. JACC: Adv.

[31] Fang N.N., Sui D.X., Yu J.G. (2015). Strain/strain rate imaging of impaired left atrial function in patients with metabolic syndrome. Hypertens Res.

[32] Kokubu N., Yuda S., Tsuchihashi K. (2007). Noninvasive assessment of left atrial function by strain rate imaging in patients with hypertension: a possible beneficial effect of renin-angiotensin system inhibition on left atrial function. Hypertens Res.

[33] Deferm S., Bertrand P.B., Churchill T.W. (2021). Left atrial mechanics assessed early during hospitalization for cryptogenic stroke are associated with occult atrial fibrillation: a speckle-tracking strain echocardiography study. J Am Soc Echocardiogr.

[34] Kusunose K., Takahashi H., Nishio S. (2021). Predictive value of left atrial function for latent paroxysmal atrial fibrillation as the cause of embolic stroke of undetermined source. J Cardiol.

[35] Chousou P.A., Chattopadhyay R., Ring L. (2023). Atrial fibrillation in embolic stroke of undetermined source: role of advanced imaging of left atrial function. Eur J Prev Cardiol.

