# Supplementary material for: Association of Left Atrial Stiffness With Risk of Cryptogenic Ischemic Stroke in Young Adults
Source: JACC Adv. 2024 Mar 14;3(4):100903. doi: 10.1016/j.jacadv.2024.100903 (PMC11198254; doi:10.1016/j.jacadv.2024.100903)
Supplement: Supplemental Table [file mmc1.docx]

**Supplemental Table 1.** Anthropometrics and clinical characteristics of CIS patients vs. controls.

|  | CIS patients (n=150) | Controls (n=150) | *p value* |
| --- | --- | --- | --- |
| Age, years | 39±8 | 39±8 | Na |
| Women | 46% | 46% | Na |
| Waist, cm | 92±14 | 90±13 | 0.156 |
| Waist-to-hip ratio | **0.89±0.09** | **0.87±0.09** | **0.046** |
| BMI, kg/m^2^ | 26.7±4.3 | 26.4±5.1 | 0.580 |
| Heart rate, bpm | **68±13** | **64±16** | **0.007** |
| Systolic BP, mmHg | **124±14** | **129±14** | **<0.001** |
| Diastolic BP, mmHg | **74±10** | **79±11** | **<0.001** |
| Hypertension | 37% | 29% | 0.142 |
| Pre-CIS antihypertensive treatment | 9% | 7% | 0.632 |
| Pre-CIS lipid-lowering treatment | 1% | 2% | 0.596 |
| Tobacco smoking | **56%** | **42%** | **0.034** |
| Physical inactivity | 9% | 5% | 0.219 |
| Heavy alcohol consumption | **21%** | **12%** | **0.033** |

BMI, body mass index; BP, blood pressure; CIS, cryptogenic ischemic stroke; Na, not applicable due to age- and sex-matching.

*P* values in column 3 indicate the level of significance when comparing patients with controls.
